# Study of influence of a model guidance about the laboratory tests and disease in knowledge and self-management of patients with type 2 DM

**DOI:** 10.1186/1758-5996-7-S1-A164

**Published:** 2015-11-11

**Authors:** Maria de Lourdes Baêta Zille

**Affiliations:** 1 SMSA/Prefeitura Municipal de Belo Horizonte, Belo Horizonte, Brazil

## Background

Diabetes mellitus (DM) has been considered a growing worldwide epidemic, with global distribution, more prevalent in developing countries. This disease causes a reduction in the quality of life of people who have it, and has brought about the increase of problems for public health systems, with extremely high social costs, especially when diagnosed late. In turn, the possibility of increasing the knowledge of the disease, in conjunction with individual behavior changes by persons with T2D, is a strategy to be considered in order for these individuals to be able to control glycemic levels more easily.

## Objective

Bearing this in mind, this study has the objective of evaluating the efficacy of the methodology of an educational program based on improving self-knowledge of diabetes, while also analyzing lab results, and treating the disease.

## Materials and methods

This study was done with 76 patients with diabetes (68.4% women and 31.6% men) registered in 12 health clinics of the central-south sanitary district of the municipal health department of Belo Horizonte. The analysis of the patients' level of knowledge before and after the implementation of the program was based on questionnaires given to patients before and after intervention.

## Results

Of exams done before and after the intervention, the following averages were obtained: Glycemia before the intervention: 161,4 mg/dL and after the interventons: 136,4 mg/dL, Total Cholesterol before the intervention: 189,4 mg/dL and after: 175,5, Triglyceride before: 160.6 mg/dL and after: 135,6 mg/dL, HbA1C before the intervention: 8.6% and HbA1C after the intervention: 7.8%.

## Conclusion

Notably, the improvement in the lab Results thus suggests the efficacy, in the context of this research, of the methodology utilized to better patients' self-knowledge of diabetes.

**Figure 1:**
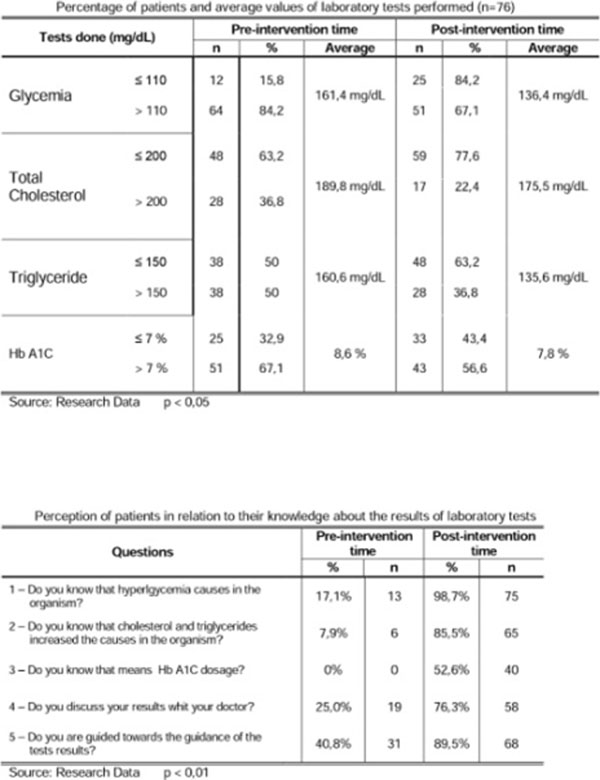
Percentage of pateints and average values of laboratory tests performs (n=76) and perception of patients in relation to their knowledge about the results of laboratory tests.

